# Age and sex differences in the effectiveness of intradialytic resistance training on muscle function

**DOI:** 10.1038/s41598-023-30621-z

**Published:** 2023-03-01

**Authors:** Aurel Zelko, Jaroslav Rosenberger, Peter Kolarcik, Andrea Madarasova Geckova, Jitse P. van Dijk, Sijmen A. Reijneveld

**Affiliations:** 1 Department of Health Psychology and Research Methodology, Faculty of Medicine, Pavol Jozef Safarik University, 040 11 Kosice, Slovakia; 2 Graduate School Kosice Institute for Society and Health, Faculty of Medicine, Pavol Jozef Safarik University, 040 11 Kosice, Slovakia; 3 Department of Community and Occupational Medicine, University Medical Center Groningen, University of Groningen, 9700 RB Groningen, The Netherlands; 4 Olomouc University Social Health Institute, Palacky University Olomouc, 771 11 Olomouc, Czech Republic; 5 2nd Department of Internal Medicine, Faculty of Medicine, Pavol Jozef Safarik University, 040 11 Kosice, Slovakia; 6 Fresenius Medical Care-Dialysis Services Kosice, 040 11 Kosice, Slovakia; 7 Institute of Applied Psychology, Faculty of Social and Economic Sciences, Comenius University in Bratislava, 821 05 Bratislava, Slovakia

**Keywords:** Renal replacement therapy, Rehabilitation

## Abstract

Previous research shows the beneficial effects of an intradialytic resistance training (IRT) on muscle function in haemodialysis patients. However, patients vary highly in their functional responses to IRT, may be due to effects of age and sex heterogeneities in adaptation. Therefore, the aim of this study was to investigate the degree to which the effects of IRT on the muscle function of haemodialysis patients vary by age and sex. We included 57 patients who completed a 12-week IRT (EXG) and 33 patients who received no IRT (CNG) during haemodialysis. Muscle function (MF) was assessed using dynamometry before and after a 12-week intervention and after a 12-week follow-up. After the 12-week intervention, we found a moderation effect of age in the relative (%) change (p = 0.011) and absolute (Δ) change (p = 0.027) of MF, and a moderation effect of sex in %MF (p = 0.001), but not in ΔMF (p = 0.069). Regarding patients’ age, the change of MF was only significantly different between EXG and CNG patients aged 60–70 years (%MF, EXG: + 34.6%, CNG: − 20.1%, p < 0.001; ΔMF, EXG: + 44.4 N, CNG: − 22.1 N, p < 0.001). Regarding patients’ sex, the change of MF was only significantly different between EXG and CNG female patients (%MF, EXG: + 23.9%, CNG: − 23.6%, p < 0.001). Age and sex did not significantly moderate changes in MF measures after 12 weeks of follow-up. We conclude that both age and sex of haemodialysis patients affect their functional response to IRT in the short term.

**Trial Registration:** Intradialytic Resistance Training in Haemodialysis Patients (IRTHEP)—#NCT03511924, 30/04/2018, https://clinicaltrials.gov/ct2/show/NCT03511924.

## Introduction

Haemodialysis and kidney disease have been shown to negatively affect patients’ physical activity behaviour, physical functioning, musculoskeletal health, body composition and quality of life in haemodialysis patients (CKD-5D)^1–3^. The participation of kidney disease patients in regular physical activities is generally low, decreasing with kidney disease progression and reaching nadir in elderly CKD-5D patients^1,2,4^. Negative trends in patient’s behaviour are manifested in decreased muscle mass and function, bone mineral density, quality of bone structure, and resulted in declined mobility, health-related quality of life, and survival rates during therapy^2–7^.

Intradialytic resistance training (IRT) positively affected physical functions, mobility, nutritional status, body composition, quality of life, dialysis-related clinical outcomes and mortality in CKD-5D patients^8–12^. IRT improved muscle functions (MF) of lower extremities, positively affects survival in CKD-5D patients, and the change in MF was associated with the presence of diabetes mellitus and microribonucleic acid expression profiles as detailed in our previous studies^13–16^. Besides clinical efficiency in the prevention of physical function decline, large inter-individual differences in the physiological response to IRT have been reported among CKD5-D patients^17–19^. The heterogeneity of findings regarding the effectiveness of IRT (on muscle functions) in CKD-5D patients may be due to individual differences in the physiological adaptation to resistance training.

Strong evidence exists for the beneficial effects of resistance training on muscle mass and function and several authors have concluded that acute responses and chronic adaptation to resistance training in healthy subjects vary by age^20–23^ and by sex^24–27^. Therefore, age and sex differences in the effects of IRT may also exist among CKD-5D patients. In the general dialysis population, the volume and function of the skeletal muscle were lower in males than in females patients, and were negatively associated with age^28–31^. No differences were found between male and female CKD-5D patients in changes in body composition, muscle size, or muscular strength, after a 12-week resistance training intervention^32^. No differences were found in the change of physical functioning between the elderly and other age groups of patients after a walking exercise intervention^33^. In summary, the current evidence regarding age and sex heterogeneities in functional adaptation in CKD-5D patients is scarce and lacking^34,35^, and recommendations for intradialytic exercise are not specified by age and sex of patients^36^. Therefore, the aim of this study was to investigate whether age and sex moderated the effectiveness of IRT in CKD-5D patients.

## Methods

### Study design

We conducted a quasi-experimental, two-group, pre-post comparative study with an intervention of 12 weeks and a 12-weeks follow-up in 2018 at three dialysis centres to assess the effects of IRT on the lower extremity MF among CKD-5D patients. A comprehensive description of the objectives, design, methods and analysis of this study is provided elsewhere^37^. The Ethics Committee of Pavol Jozef Safarik University in Kosice reviewed and approved the study protocol (Approval no. 14N/2017). All methods, assessments and data acquisition were conducted in accordance with the Declaration of Helsinki of 1975 and with the Good Clinical Practice Principles of the International Council for Harmonization. The study was registered in ClinicalTrials.gov on 30/04/2018 (NCT03511924).

### Subjects

For the purpose of this study we assessed the eligibility of patients treated at three dialysis centres (two dialysis centres located in Kosice, one dialysis centre located in Banska Bystrica). We selected three centres to meet the expected patient numbers in the study groups, and took specifically these three because they had identical patients' treatment regimens and a similar age and gender distribution. The inclusion criteria were as follows: age above 30 years, diagnosed with stage 5 chronic kidney disease, history of maintenance dialysis therapy for at least the last 3 months. Exclusion criteria were lower extremity amputation, severe dementia or retardation, presence of acute intercurrent disease and the probability of 1-year mortality higher than 25% according to the Charlson Comorbidity Index^38^. Informed consent was obtained from all individual participants included in the study.

### Sample size calculation

For the purpose of this study, its statistical power was re-calculated with use of GPower 3.1^®^ (Heinrich-Heine-University, Düsseldorf, Germany). We used a priori F test for an analysis of variance, with ten subgroups (two interventions by three age and two sex categories), a power of 80% and an effect size (Cohen’s *f*) of 0.40. We found that at least 64 patients totally are needed to detect differences in the change in MF by the intervention, age and sex.

### Patient allocation

Patients attending dialysis therapy at both sites in Kosice were allocated to the experimental group (EXG, n = 57), while patients from the Banska Bystrica dialysis centre were allocated to the control group (CON, n = 33). After the allocation procedure, the investigatory team members and participating patients were informed about the group assignment structure^16^.

### Intervention period—experimental condition

All EXG patients started the 12-week IRT programme according to clinical recommendations for exercise interventions in CKD-5D patients within a week after completion of the baseline assessments^36^. EXG subjects were asked to follow the prescribed IRT programme and not to make any significant lifestyle regimen and exercise behavioural changes during the time of the study, especially in the RT component. A detailed description of the methodology, periodization and progressivity of IRT applied in EXG patients is provided elsewhere^37^.

### Intervention period—control condition

Patients allocated to the CNG received their standard nephrology care without any intervention increasing their physical activity during dialysis. These patients were requested to maintain their standard treatment regimen and to maintain their customary dietary and physical activity patterns, especially in the RT component. During the control period these patients received increased attention from the research team members but were physically inactive during haemodialysis sessions.

### Follow-up period

All patients enrolled in the study underwent a 12-week follow-up period after the completion of the experimental or control condition. During the follow-up, the patients were instructed not to participate in any structured physical activity during dialysis.

### Measures—primary outcome

We used muscle function (MF) a primary outcome, measured as the maximal force produced by the patient during isometric contraction of hip extensor muscles. During the assessments, patients were in a supine position with arms safely and comfortably placed on the bed. The patient held the dominant leg in a straightened position, while the dynamometer was placed proximally to the ankle on the posterior surface of the lower leg. The patients were instructed to perform a maximal isometric contraction and hold it for 5 s. The tests were repeated within 30-s rest intervals, and the higher measured values of two consecutive tests were used for the analysis.

Before the assessments of MF took place, patients became familiar with test protocol and realized an exploratory set of the patient’s MF assessments with emphasis on the proper execution of muscle contractions. At the consecutive dialysis session, maximal isometric contraction force during the extension of the lower limb at the hip joint was assessed using a hand-held dynamometer (Universal digital force gauge HF 500, SAUTER GmbH, Balingen, Germany). The range of the dynamometer analyser was set from 0 to 500 N, with a recording interval of 0.1 N. These assessments of maximal isometric contraction force have excellent interrater reliability and accuracy^39–41^. The accuracy of the device used for assessments of muscle function in our study was verified with standard weights and the margin of error was below 5%. The absolute changes (ΔMF) of maximal isometric forces were calculated as post-intervention measure minus baseline measure and post-follow up measure minus baseline measure. The relative changes (%MF) of maximal isometric forces were calculated as the absolute value of post-intervention and post-follow up changes divided by baseline measure and multiplied by 100. All physical tests were administered by one member of the investigatory team (AZ).

### Measures—background variables

We collected clinical measures (values registered from the last preceding serology and haematology tests) and body composition (patient’s body weight and height) from the medical database. We calculated the body mass index (BMI) for all patients as body weight in kilograms divided by the body height in metres squared (kg/m^2^). Patients’ age in decimals of years and sex (male/female) were collected from the medical database. For the analysis of differences between age groups, we categorised patients younger than 60.0 years in the middle-aged patients group (MA). Patients with an age between 60.0 and 70.0 were categorised in the younger-old group (YO), and patients older than 70.0 years were categorised in the older-old group (OO).

Background variables regarding the physical activity behaviour were assessed during an investigator-patient interview before and after exposure to the experimental and control conditions^13^. Regarding the individual physical activity we measured patient-reported frequency, duration and type of physical activities following the instructions of the Global Physical Activity Questionnaire^42^. A patient was considered to be physically inactive if he or she reported less than 3 × 30 min of moderate-intensity physical activity per week^43^.

### Statistical analysis

First, we used the Kolmogorov–Smirnov test to assessed data normality, and the Levene’s test to analyse the homogeneity of variances in our database. Second, we assessed and quantified the flow and losses of subjects through the intervention and follow-up period of the study and briefly reported reasons for dropouts in each phase of the study according to the CONSORT statement recommendations^44^. Third, we assessed baseline primary outcome and background variables and compared them between the age and sex groups in EXG and CNG patients by one-way analysis of variance test. Data were presented as mean (M) ± standard deviation (SD). Fourth, we assessed whether effects of the experimental and control condition on the primary outcome (ΔMF and %MF) is moderated by patient’s sex or age, directly after the intervention and after the follow up period. We did so by adding these variables as moderator of group allocation in generalized linear model (GLM), and assessing the overall improvement of model fit based on that. We used the univariate GLM with the Bonferroni corrections for multiple comparisons to test main effects of allocation, age and sex (fixed factors), and moderation effects of allocation and age; and allocation and sex on the primary outcome (dependent value). Bonferroni post hoc tests were used to localize differences between the patients’ allocation and age or sex groups. Estimates of effects were presented as mean differences with 95% confidence interval (95% CI). We performed the analyses on an intention-to-treat basis, i.e., always including all 90 patients who had been enrolled in the study and had completed the baseline assessments regarding the primary outcomes. Statistical significance was defined as a *p* value below 0.05. Data analyses were carried out using the statistical software package IBM SPSS 22.0 (Version 22.0. Armonk, NY: IBM Corp.).

## Results

### Patient flow

We screened all 198 patients of three dialysis centres and regarding the inclusion and exclusion criteria, through their nephrologists. We identified 126 eligible patients and informed them about the possibility to participate in the study. In the end, 90 patients agreed to participate and signed a written informed consent prior to the study. Patients treated at two dialysis centres located in Kosice were allocated to the experimental group (EXG, n = 57). Patients treated at the dialysis centre in Banska Bystrica were allocated to the control group (CNG, n = 33).

**Figure 1:**
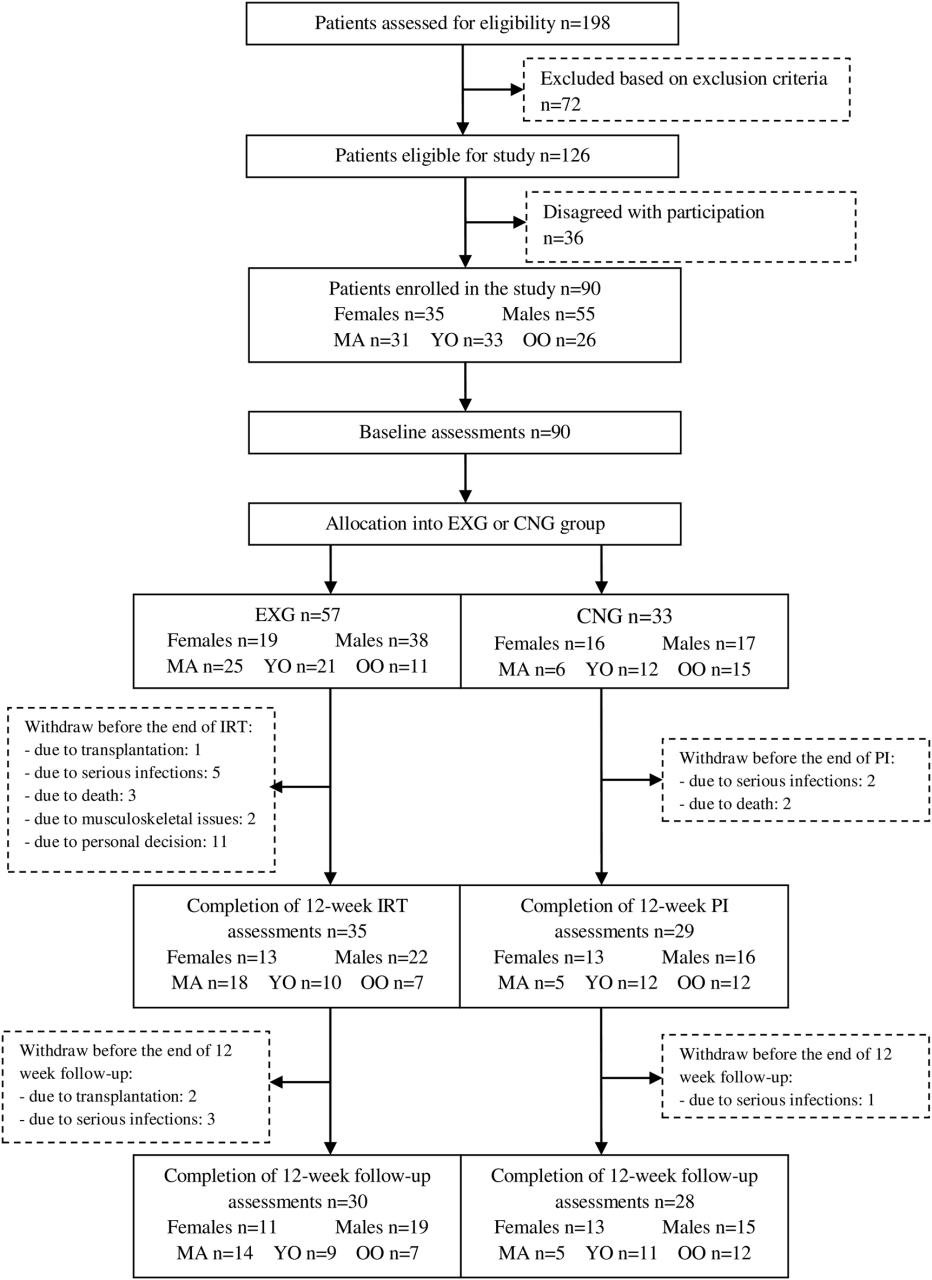
The CONSORT flow diagram of patients summarising patients’ eligibility assessment, enrolment and allocation into the experimental (EXG) and control group (CNG) of the study and distribution of patients regarding age and sex subgroups (*EC* experimental condition, *CC* control condition, *MA* middle-aged, *YO* younger-old, *OO* older-old group).

From the 57 patients initially included in the EXG, 22 patients discontinued participation in the study during the experimental condition. From the 33 patients who initially entered the CNG, four patients discontinued participation in the study during the control condition. During the 12-week follow-up, five patients in the EXG and one patient in the CNG dropped out due to mortality, transplantations, serious infections, personal decisions, and musculoskeletal issues. No adverse effects occurred during the application of exercise interventions or muscle strength assessments; see further the CONSORT flow diagram (Fig. 1)^44^. The resultant statistical power (1 − β error probability) of the study sample included in the data analysis was 0.93.

### Characteristics of the study participants

Patients’ baseline characteristics enrolled in the EXG and the CNG arm by age and sex are presented in Tables 1 and 2, respectively. The baseline assessments of the physical activity behaviour showed that 83 patients (92%) did not participate in customary physical activities.

**Table 1:** Baseline patient characteristics, by arm and age.

Variable	Experimental condition EXG (n = 57)				Control condition CNG (n = 33)			
	MA (n = 25)	YO (n = 21)	OO (n = 11)	p value	MA (n = 6)	YO (n = 12)	OO (n = 15)	p value
Age in years (M, SD)	47.6 (10.2)	65.5 (3.3)	76.2 (3.7)	< 0.001*	51.9 (5.1)	65.2 (3.1)	75.6 (4.5)	< 0.001*
Body weight in kg (M, SD)	77.9 (13.0)	81.8 (20.3)	68.7 (15.5)	0.111	63.3 (8.1)	75.5 (12.4)	68.0 (17.2)	0.202
Body mass index in kg/m^2^ (M, SD)	26.6 (5.2)	28.1 (6.7)	25.2 (4.7)	0.384	22.8 (3.3)	26.2 (4.2)	24.2 (5.5)	0.326
Dialysis adequacy in Kt/V (M, SD)	1.5 (0.3)	1.6 (0.4)	1.6 (0.4)	0.859	2.0 (0.3)	1.9 (0.4)	2.1 (0.3)	0.499
Over-hydration index in % (M, SD)	11.8 (9.6)	12.2 (5.6)	12.0 (3.5)	0.983	9.9 (7.2)	14.3 (5.0)	11.3 (7.8)	0.360
C-reactive protein in mg/l (M, SD)	10.3 (14.7)	12.6 (13.8)	11.7 (5.2)	0.837	2.9 (3.5)	8.4 (9.2)	12.3 (14.3)	0.236
iPTH in pg/ml (M, SD)	495.4 (436.9.0)	362.9 (281.3)	196.2 (113.7)	0.057	186.2 (114.3)	467.5 (560.4)	370.7 (338.9)	0.407
Haemoglobin in g/l (M, SD)	112.0 (14.2)	112.0 (11.3)	112.2 (14.7)	0.999	114.8 (5.9)	111.8 (17.0)	113.2 (13.9)	0.913
Albumin in g/l (M, SD)	39.4 (2.6)	39.1 (3.2)	39.6 (2.0)	0.882	37.3 (1.0)	35.8 (5.7)	37.6 (4.4)	0.567
Ferritin in ng/ml (M, SD)	558.8 (451.7)	609.3 (466.7)	649.6 (749.3)	0.881	878.5 (355.1)	841.4 (358.1)	834.9 (352.8)	0.967
Phosphates in mml/l (M, SD)	1.8 (0.5)	1.6 (0.3)	1.9 (0.5)	0.078	1.4 (0.3)	1.5 (0.6)	1.4 (0.4)	0.710
Calcium in mmol/l (M, SD)	2.2 (0.2)	2.2 (0.2)	2.0 (0.3)	0.128	2.3 (0.1)	2.2 (0.1)	2.4 (0.1)	0.367
Potassium in mEq/l (M, SD)	5.1 (0.7)	5.0 (0.6)	5.5 (1.0)	0.167	5.5 (0.6)	5.1 (0.8)	5.1 (1.0)	0.583
Sodium in mEq/l (M, SD)	138.2 (3.8)	138.0 (2.4)	138.0 (2.9)	0.949	138.7 (2.7)	137.8 (2.2)	138.6 (2.9)	0.664
Hip extension in N (M, SD)	178.6 (55.0)	171.3 (66.3)	113.7 (53.4)	0.011^**#**^	160.3 (57.0)	147.8 (52.6)	133.8 (36.5)	0.476

*iPTH* intact parathyroid hormone, *N* Newton, *EXG* experimental group, *CNG* control group, *MA* middle-aged, *YO* younger-old, *OO* older-old group. Data are presented as mean (M) ± standard deviation (SD), *p* values determined by analysis of variance tests. ^**#**^Differences between groups significant at p < 0.05. *Differences between groups significant at p < 0.001.

**Table 2:** Baseline patient characteristics, by arm and sex.

Variable	Experimental condition EXG (n = 57)			Control condition CNG (n = 33)		
	Females (n = 19)	Males (n = 38)	p value	Females (n = 16)	Males (n = 17)	p value
Age in years (M, SD)	64.6 (14.2)	57.3 (12.6)	0.055	69.2 (10.1)	65.9 (9.4)	0.332
Body weight in kg (M, SD)	75.2 (19.6)	78.8 (15.5)	0.449	64.7 (14.1)	74.7 (13.8)	0.046^**#**^
Body mass index in kg/m^2^ (M, SD)	28.5 (7.3)	26.1 (4.6)	0.122	24.6 (5.2)	24.8 (4.5)	0.932
Dialysis adequacy in Kt/V (M, SD)	1.8 (0.3)	1.5 (0.3)	0.001^†^	2.2 (0.3)	1.8 (0.3)	0.002^†^
Over-hydration index in % (M, SD)	10.4 (4.5)	12.7 (8.3)	0.260	12.3 (7.4)	12.0 (6.4)	0.923
C-reactive protein in mg/l (M, SD)	8.1 (4.6)	13.1 (15.3)	0.175	9.1 (13.2)	9.3 (10.1)	0.959
iPTH in pg/ml (M, SD)	480.7.8 (424.3)	342.9 (308.4)	0.167	491.8 (554.6)	259.9 (155.8)	0.108
Haemoglobin in g/l (M, SD)	109.8 (12.8)	113.2 (13.3)	0.361	108.1 (10.4)	117.6 (15.3)	0.048^**#**^
Albumin in g/l (M, SD)	38.8 (3.1)	3739.6 (2.5)	0.317	36.2 (4.9)	37.5 (4.1)	0.403
Ferritin in ng/ml (M, SD)	695.2 (544.8)	627.6 (452.0)	0.304	932.4 (248.1)	763.1 (405.6)	0.161
Phosphates in mml/l (M, SD)	1.7 (0.4)	1.8 (0.5)	0.679	1.5 (0.5)	1.5 (0.4)	0.905
Calcium in mmol/l (M, SD)	2.1 (0.2)	2.2 (0.3)	0.387	2.4 (0.1)	2.3 (0.1)	0.003^**†**^
Potassium in mEq/l (M, SD)	5.4 (0.8)	5.0 (0.7)	0.056	5.1 (1.0)	5.3 (0.6)	0.489
Sodium in mEq/l (M, SD)	138.6 (3.5)	137.8 (2.9)	0.354	138.6 (2.0)	138.1 (3.1)	0.589
Hip extension in N (M, SD)	113.1 (52.2)	188.5 (52.5)	< 0.001*	122.0 (32.6)	164.1 (48.7)	0.007^**†**^

*iPTH* intact parathyroid hormone, *N* Newton, *EXG* experimental group, *CNG* control group. Data are presented as mean (M) ± standard deviation (SD), *p* values determined by analysis of variance tests. ^#^Differences between groups significant at p < 0.05. ^†^Differences between groups significant at p < 0.01. *Differences between groups significant at p < 0.001.

### Differences in effects of 12-week intervention period on the primary outcome by age and sex

After 12-week intervention, we found a significant effect of the intervention on %MF (η^2^ = 0.199, p = 0.001,) and ΔMF (η^2^ = 0.137, p = 0.004). Effects on both measures of MF were significantly greater in the EXG compared to the CNG group (%MF: difference 25.2%, 95% CI = 11.8 to 38.6%, p = 0.001; ΔMF: difference 28.9 N, 95% CI = 9.7–48.1 N, p = 0.004)*.*

**Figure 2:**
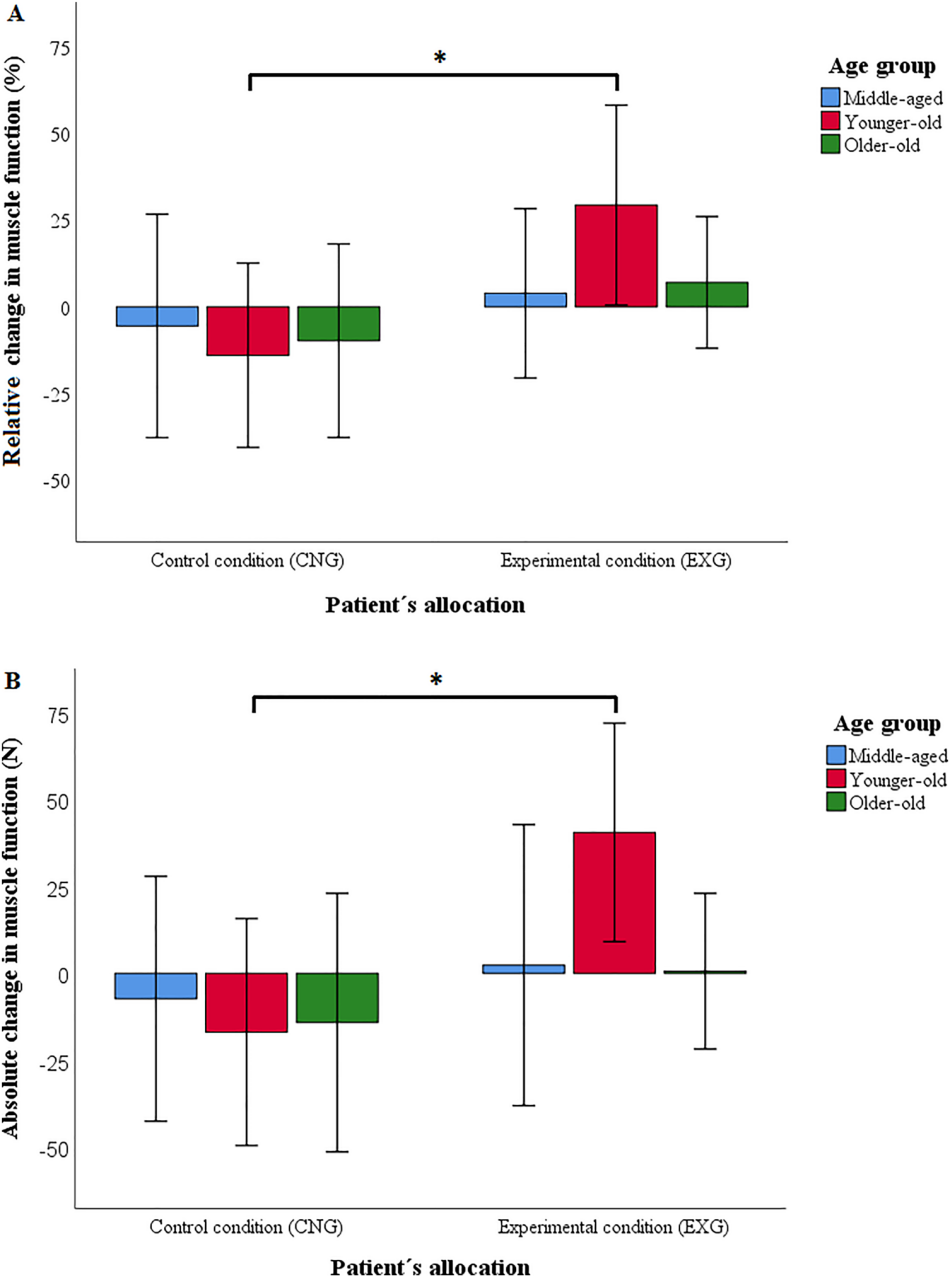
Relative (**A**) and absolute (**B**) changes in muscle function by patients’ allocation and age after the 12-week intervention (*CNG* control condition, *EXG* experimental condition). Data were presented as mean ± standard deviation. *Differences between groups significant at p < 0.001. p values calculated for intention-to-treat analysis (n = 90).

We found a significant effect of age on the effect of the intervention on of %MF (η^2^ = 0.145, p = 0.011) and on ΔMF (η^2^ = 0.119, p = 0.027). Both measures of MF change differed significantly between EXG and CNG in YO patients (%MF: difference = 54.7%, 95% CI = + 32.2 to + 77.1%, p < 0.001; ΔMF: difference = 66.5 N, 95% CI = + 34.3 to + 98.7 N, p < 0.001; see Fig. 2A,B). However, they did not differ in MA (%MF: difference = 10.2%, 95% CI = − 14.5 to + 35.0%, p = 0.441; ΔMF: difference = 9.9 N, 95% CI = − 25.5 to + 45.4 N, p = 0.577; see Fig. 2A,B) and neither in OO patients (%MF: difference = 10.8%, 95% CI = − 12.8 to + 34.4%, p = 0.364; ΔMF: difference = 10.2 N, 95% CI = − 23.6 to + 44.0 N, p = 0.548; see Fig. 2A,B).

**Figure 3:**
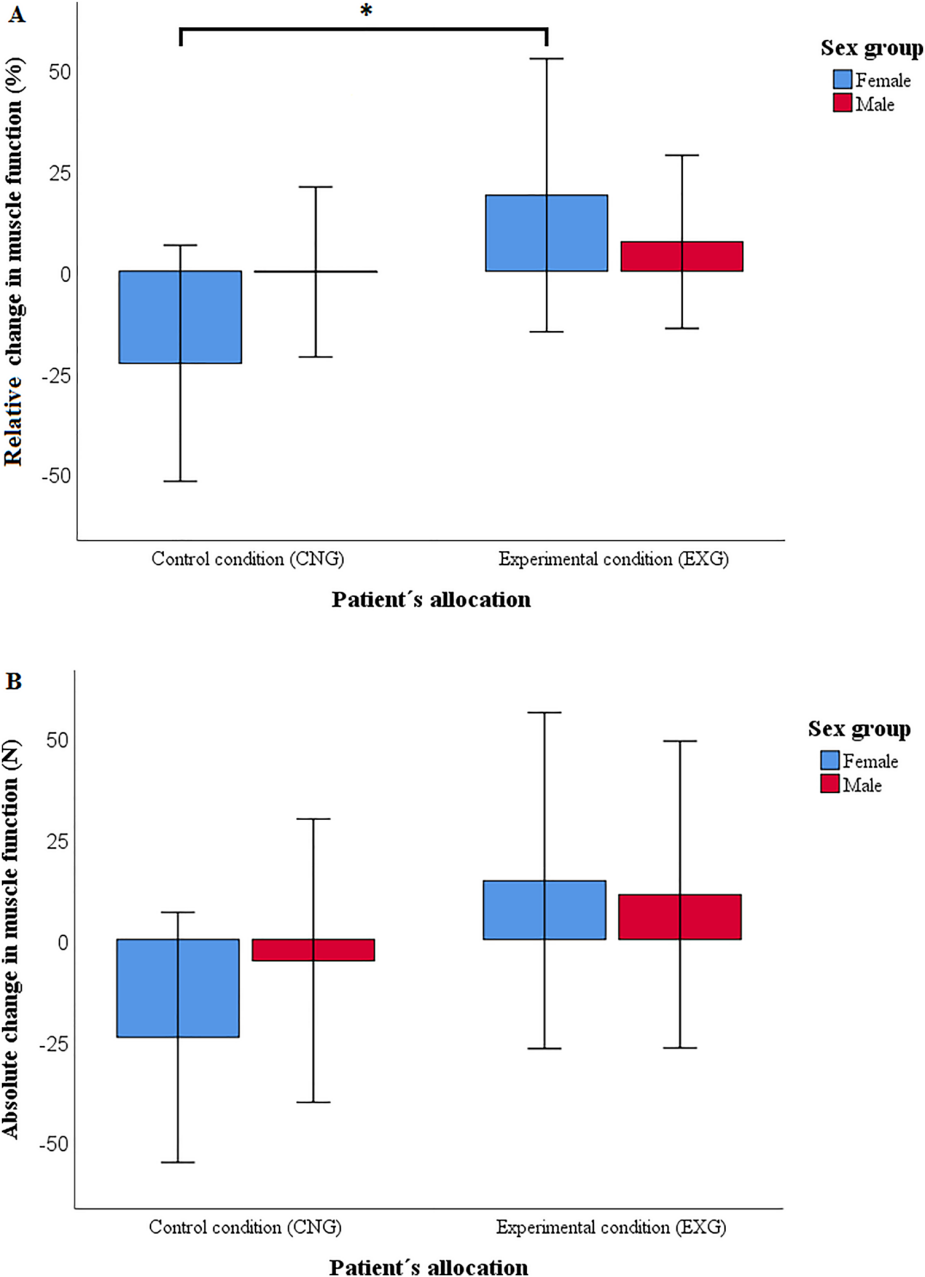
Relative (**A**) and absolute (**B**) changes in muscle function by patients’ allocation and sex after the 12-week intervention (*CNG* control condition, *EXG* experimental condition). Data were presented as mean ± standard deviation. *Differences between groups significant at p < 0.001. p values calculated for intention-to-treat analysis (n = 90).

We found a significant effect of sex on the effect of the intervention in the change of %MF (η^2^ = 0.165, p = 0.001), however not for change of ΔMF (η^2^ = 0.057, p = 0.069). The %MF change differed significantly between EXG and CNG female patients (%MF: difference = 47.5%, 95% CI = 27.7 to 67.3%, p < 0.001), but not between EXG and CNG male patients (%MF: difference = 3.0%, 95% CI = − 14.9 to + 20.8%, p = 0.741, see Fig. 3A,B). Differences in the changes of %MF and ΔMF in the CNG and EXG patients, by age and sex, are presented in Tables 3 and 4, respectively.

**Table 3:** Comparison of differences in relative change of muscle function and 95% confidence intervals (CI) from baseline to first post-measurement, between the experimental and control group, regarding patient’s age and sex.

Group	Middle aged	Younger old	Older old	Female	Male
EXG	+ 7.4 (5.7)	+ 34.6 (8.2)	+ 3.2 (9.4)	+ 23.9 (7.0)	+ 6.2 (5.7)
CNG	− 2.8 (10.9)	− 20.1 (7.7)	− 7.6 (7.1)	− 23.6 (7.1)	+ 3.3 (6.9)
Mean difference	10.2 (12.3)	54.7* (11.2)	10.8 (11.8)	47.5* (9.9)	3.0 (8.9)
95% CI	− 14.5 to + 35.0	+ 32.2 to + 77.1	− 12.8 to + 34.4	+ 27.7 to + 67.3	− 14.9 to + 20.8

Data are presented as means (relative changes in muscle function) and standard deviations. EXG, experimental group; CNG, control group. p-value calculated for intention-to-treat analysis (n = 90, nEXG = 57, nCNG = 33). Difference between groups significant at p < 0.001 is marked by *.

**Table 4:** Comparison of differences in absolute change of muscle function and 95% confidence intervals (CI) from baseline to first post-measurement, between the experimental and control group, regarding patient’s age and sex.

Group	Middle aged	Younger old	Older old	Female	Male
EXG	+ 4.9 (8.2)	+ 44.4 (11.7)	− 2.1 (13.5)	+ 22.0 (10.0)	+ 9.4 (8.2)
CNG	− 5.1 (15.7)	− 22.1 (11.0)	− 12.3 (10.2)	− 24.5 (10.1)	− 1.8 (9.8)
Mean difference	9.9 (17.7)	66.5* (16.1)	10.2 (16.9)	46.5 (14.2)	11.2 (12.8)
95% CI	− 25.6 to + 45.4	+ 34.3 to + 98.7	− 23.6 to + 44.0	+ 18.1 to + 75.0	− 14.4 to + 36.9

Data are presented as means (absolute changes in muscle function) and standard deviations. EXG, experimental group; CNG, control group. p-value calculated for intention-to-treat analysis (n = 90, nEXG = 57, nCNG = 33). Difference between groups significant at p < 0.001 is marked by *.

### Differences in effects of 12-week follow-up on the primary outcome by age and sex

We did not find a significant effect of age (%MF: η^2^ = 0.105, p = 0.053; ΔMF: η^2^ = 0.075, p = 0.128), and neither of sex (%MF: η^2^ = 0.056, p = 0.082; ΔMF: η^2^ = 0.043, p = 0.127), on the effect of 12-week follow-up in the changes of %MF and ΔMF.

## Discussion

IRT positively affects MF in CKD-5D patients. We found that the beneficial effects of IRT on MF manifested only in YO and female CKD-5D patients.

We found that the intervention had effects on %MF and ΔMF in YO patients, but did not find such differences in effects on MF measures for MA and OO patients. A possible explanation for this age-dependency of effects may be that the prescribed IRT and its progressivity were really suited for YO patients but did not suit for MA and OO patients. In the OO patients, the relatively frequent cardiovascular and metabolic comorbidities may have lowered their functional adaptability to IRT^45,46^. This partially aligns with some previous reports on CKD-5D patients that found beneficial effects of exercise on MF in patients aged above 60 and 65 years^33,47,48^ and with reports of no improvements in lower extremity muscle strength after intradialytic exercise in patients aged above 70 and above 80 years^49,50^. However, other studies reported no age-differences in functional adaptation after intradialytic- and home-based exercise in CKD-5D patients^19,44^. A first explanation of these discrepancies might regard the different shares of age groups in the various studies. The studies that reported no age differences included experimental subjects with a mean age 68 ± 13 years, and 72 (69–79) years; and control subjects in mean age of 68 ± 11 years, and 76 (69–78) years, respectively^19,44^*.* In our study, we included experimental subjects with a mean age of 60 ± 13 years and control subjects with a mean age of 68 ± 10 years. This lower average age of our patients might be a source of the contrasting conclusions. A second explanation regards the type of physical activity intervention as assessed. The studies that reported no age-heterogeneity used a combination of aerobic and resistance exercise as the intervention. In contrast, we applied resistance training as the intervention which may be more effective in CKD-5D patients.

We found that the intervention had effects in female patients, but not in male patients. This is in contrast with the findings of previous studies on changes in MF among patients with chronic kidney disease, which reported no sex-related differences in MF change^51^ and beneficial effects in male dialysis patients but not in female^52^. A reason could be that we included patients diagnosed with stage 5 CKD on maintenance haemodialysis therapy, whereas the previous study included stage 4 and 5 CKD patients who were on pre-dialysis therapy^51,52^. The higher severity of the disease and application of maintenance haemodialysis therapy in patients enrolled in our study might have contributed to different conclusions regarding the role of patients’ sex in functional adaptation. Alternatively, this may simply be a chance finding, given that one study reports no sex differences, a second one effectiveness in males and ours effectiveness in females. Evidently, this requires further study.

This study has several important strengths. First, we carried out procedures during the haemodialysis therapy, which enabled us to obtain more realistic data in a reproducible design. Second, the allocation of patients into the EXG and CNG groups according to the geographical location of care-providing dialysis centres minimised the likelihood of contamination of the CNG subjects by the intervention throughout the study.

Our study also has some limitations, however. First, we used a quasi-experimental design with the allocation of patients into arms based on the dialysis centre location, which may have led to differing samples per arm. However, to control baseline imbalances between subgroups in body weight, dialysis adequacy, haemoglobin, calcium, we analysed differences in primary outcome measures using models adjusted for these patients’ characteristics. This hardly affected our findings. Second, assessors of the outcome measures were not blinded, which may have caused information bias. However, the assessments were highly standardised, limiting the potential effect of this. Third, we used hand-held dynamometry for MF measures assessments. Compared to isokinetic dynamometry, handheld dynamometry is a less reliable diagnostic instrument, which may have produced differences in MF assessments between male and female healthy subjects, potentially leading to bias^53^. However, another study reported a high reliability and validity for the assessment of MF by handheld dynamometry in female and male CKD-5D patients^54^. Fourth, we did not assess the muscle quantity in CKD-5D patients, and therefore we were not able to report associations between observed age- and sex-related heterogeneity in the change of MF measures and muscle tissue structure indicators. Fifth, the proportion of YO female patients was lower in EXG (15%) compared to CNG (23%), and the proportion of YO patients was lower in females (19%) compared to males (41%). However, we found that all reported differences between subgroups were not statistically significant.

Patients’ age and sex play an important role in the response of MF to IRT. We found that the IRT is much more effective in YO patients and in female patients, implying special attention is needed for organisation of exercise interventions for MA and OO patients and male patients. This implies that age and gender should be considered regarding individual intensity, duration and frequency prescriptions for RT. Regarding other implications of our results for clinical practice, our study provided interesting evidence about assessment of muscle function among CKD-5D patients. The associations between patients’ physical functions, mortality and importance of physical performance assessments in CKD-5D patients are well described^55–57^. However, assessment methods of physical function applied in clinical practice differs in applicability and accuracy^58^. We found different effect sizes for age and sex between the measures of %MF and ΔMF. Both measures of MF change were moderated by allocation and age, however only %MF was moderated by allocation and sex. It may be assumed that both calculation methods of MF changes are feasible for CKD-5D patients; however assessment of patients’ %MF may provide more sex-specific information on functional adaptation after physical interventions.

Future research might focus on the effectiveness of exercise prescriptions tailored to the CKD-5D patients’ characteristics^2,19,36^. Furthermore, the functional assessments for age and sex heterogeneity analyses in CKD-5D patients might be realized by isokinetic dynamometry and after the application of other types of exercise and nutritional interventions^46^.

## Data Availability

The datasets generated during and/or analysed during the current study are available in the ZENODO repository, at: 10.5281/zenodo.7019159; reference number: 7019159.
